# Microscopical Examination of Spots of Blood

**Published:** 1858-02

**Authors:** 


### ECLECTIC DEPARTMENT,

### AND SPIRIT OF THE MEDICAL PERIODICAL PRESS.

---

*Memoir on the Medico-Legal Examination of Spots of Blood, by means of the Microscope.* By Dr. CHARLES ROBIN, Professor of the Faculty of Paris, etc., etc.

In all medico-legal researches, learned men have turned their attention almost exclusively to the chemical tests, and attached much importance to variations in the proportionate qualities of the different elements contained in the matters submitted to analysis.

This method could only give results so vague and uncertain, that no positive conclusions could be drawn from them. In order that medical science should not fail in its mission, when justice appeals to it for instruction and guidence, it is necessary that we should have recourse to other means than those furnished by chemical analysis, and mere external appearance of the spots.

With this hope we have made use of the microscope, and we can confidently expect from it the most useful results, as it has already cleared more than one point in legal medicine.

It may not be unnecessary to state for those who have not turned their attention to the recent applications of this instrument, that the microscope by its very physical construction shows, and can only show, what actually exists.

All organized tissues are composed of particles infinitely small, invisible to the naked eye, called anatomical elements, the well known characters of which enable us to distinguish them from each other at the different periods of intra and extra-uterine life. On the other hand, the various liquids of the animal economy contain certain elementary particles peculiar to them, and only visible by means of a high magnifying power, but they also carry along some particles of the mucous membrane over which they may happen to pass, such as the many kinds of epitheliums which are constantly renewed, and which differ from each other accordingly in different regions of the body, thus allowing their origin to be easily recognized.

The structure and disposition of the anatomical elements of the body, being once known, they become an excellent means of deciding as to the nature of any of the tissues or liquids of the animal economy; and therefore it is, that an examination through the medium of the microscope, gives more positive results than other methods of investigation.

In fact, this instrument shows us the constituent and organized portions of those tissues, and not the reäctions produced by their chemical decomposition, according to the method heretofore employed. The constituent particles of any body, whether of organic or vegetable origin, cannot be mistaken for the elementary forms of animal structure, whether we examine their form, their chemical properties, or their physical appearance; but their chemical analysis is far from offering the same absolute certainty.

The great advantage of microscopic examination, as will appear from the facts hereafter cited, is that however small be the quantity of matter submitted for legal investigation, it may nevertheless suffice for demonstrative evidence by means of the microscope. Thus, where chemistry would be at a loss for want of sufficient quantity to be operated on by its tests, there the microscopic examination would be perfectly satisfactory: such, for instance, as epidermic scales, or portions of fatty matter.

The application of this means of investigation has no other limits but those of nature herself, and every portion of the human body may thus be scientifically recognized: for blood, bile, meconium, fœcal matter, vomiting, the brain, the skin, the muscles, fat, etc., have all their distinctive anatomical elements, which the microscope can recognize with unfailing certainty. Nor is it necessary to extend this enumeration of the possible applications of this instrument, for independently of the tissues of the body it may be most useful in determining the identity of any small portion of clothing, the nature of its texture, whether animal or vegetable, and numerous other problems, the solution of which is not only susceptible of the utmost accuracy, but will become comparatively easy for any one acquainted with the structural anatomy of plants and vegetables, by means of the microscope.

We hope that the application of this means to the particular case in hand, will illustrate still farther the nature and bearing of the preceding considerations.

The examination of the stains of blood is one of the most delicate questions in legal medicine. When chemical tests are used for this investigation, as has been the case heretofore, it is impossible to arrive at any certain or determinate result, but only a mere approximation, the most evident reason of which is, that chemistry examines the proximate elements of the blood, such as albumen and fibrine, and not the essential elements, such as the red and white globules. Microscopical investigation, combined with the different chemical agents, must offer, therefore, the highest degree of cumulative evidence, and the details of the following case will afford abundant proof of this assertion.

In the following pages we shall confine our attention to the description of the anatomical elements, which characterize the blood; but we must now observe that microscopical investigation of the different liquids, though sufficiently accurate, is not so absolutely certain as that of the tissues themselves, of the human body. Their anatomical elements, though invisible to the naked eye, can yet be easily recognized by means of the microscope; on the other hand, they are not so susceptible of characters at different ages of intra and extra-uterine life. Thus it is that small portions of the fœtal membranes of the placenta, of the deciduous membrane, of the clots formed in the womb can all be easily recognized; that the beard, the hair of man, and other animals, the fatty tissue, the nervous matter, etc., can be easily determined, and these are the very cases in which chemical tests are most unsatisfactory.

Here is the text of the instructions directed to Dr. Salmon, Surgeon of the Hotel Dieu, of Chartres, and myself, in the case of the murder of Louvet, by Gontier. [Extract from a Memoir published by Dr. Charles Robin, in the Annals of Hygiene and Legal Medicine, vol. xliv, p. 190.]

*Whereas*, This blouse, soiled as it is, was seized eight days after the crime, has not been washed since the murder, and has retained several spots of blood which the accused admits to be such.

*Whereas*, These spots of blood are found not only on the sleeves and front of this garment, but on the back, and that some of these spots, especially those on the sleeves and the shirt, appear to have been rubbed either with water or earthy matter.

*Whereas*, The accused has endeavored to explain these spots by saying that they were caused by blood which sprung from a duck killed in his presence.

*Whereas*, Although the circumstance which he invokes be not correct, and that the duck in question was not killed in his presence, it is important, nevertheless, to ascertain whether the spots of blood which are found on this blouse be really the blood of a duck, admitting the blood thus derived could have been projected in sufficient quantity to explain the numerous spots which exist on the sleeves, on the front, on the shoulder, and on the back, and whether in the second place, it is not more probable that this blood may be proved by its intrinsic characteristics to be that of an old woman who was violently struck on the head by means of a sharp, and heavy instrument.

*Whereas*, In such a case the microscope is not used by scientific men as a means of accurate investigation. We therefore submit the following questions to the two Doctors appointed as experts in the case:

1. Are the spots on the blouse, especially those of a dark color, with a brownish yellow appearance, really blood (and the experts shall not only say whether it contains the albuminous elements of the blood, but whether it be what really constitutes blood)?

2. Independently of the spots which appear like blood, even to the naked eye, are there not, on the blouse, other spots of the same nature, but not so dark, which must have been washed or rubbed with the intention of effacing them?

3. Are these spots of blood in such quantities and so differently situated that they cannot be explained by the splashing of a fowl killed in the presence of a man, wearing this blouse, and having his face turned towards the bird?

4. These elements of blood cognizable by the microscope, could they belong to a duck, which on being killed, may have thrown that blood in different directions?

5. On the other hand, are not these component particles of blood, such as belong to the human species? and if possible, designate the probable age of the person from whom they were derived.

6. If those spots be of human blood, may they not have been produced by the act of a person striking another with a hatchet or a spade?

7. The said experts will explain in their reply, their method of investigation, the guarantees, the security, and accuracy, offered by the processes they have adopted for the execution of their mandate.

Sec. II. *Examination of the Spots by the naked Eye, and a simple Magnifying Glass.*

In order to answer the above questions we have proceeded as follows:

After having counted and measured the spots of blood, we found that they varied in size from a quarter of a millimetre to three and a half millimetres; their nature, viz., blood, could be easily known by their color of a dark red, by day light, and of a brilliant black by lamp light, reflecting the latter with that peculiar vividness which is characteristic of spots of blood observed in those conditions. But this mode of reflecting the light from a candle or a lamp, is also a property of egg albumen of gelatine of gum, and probably of all liquids rich in albuminous principles. But the dark red color joined to this brilliant refraction of light is already sufficiently characteristic to guide us in the different methods of investigation.

All of these spots when examined by means of a simple magnifying glass, showed us a crust, slightly salient above the tissue of the cloth; each crust was brilliant under certain directions of light, but dark and opaque at other angles of inclination. These crusts were so very thin that it was impossible to appreciate them by the naked eye; they measured from one to two millimetres in length.

The smallness of the spots, the thinness of the crust by which they were formed, soon satisfied us of the utter impossibility of using the tests employed for an examination of the coloring matter of the blood, and its albumen, in order to decide as to their nature.

But the small black crust on the tissue of the blouse allowed us to decide positively as to the existence of the essential particles of the blood on each of the spots, respectively, notwithstanding their small extent. Those which reached the size of three millimetres and a half, were divided into two parts, by cutting the cloth in order that each portion might be submitted to a comparative examination by somewhat different methods.

§ III. *Examination by means of the Microscope of the Spots whose appearance to the naked Eye afforded sufficient presumption of their being Spots of Blood.*

With a certain number of the spots of which we have given the external characters, and having divided into two parts the largest of these, as above related, we proceeded in the following manner:

We cut into slips the cloth on which were seen two of the spots as above described, and immersed it during six or eight hours in pure water. To do this, the lower extremity alone of the strip containing the spot was plunged in water until it approached within two or three millimetres of the spot it self, which was out of water, but resting against the upper portion of the capsule holding the water. The fluid soon rises by capillary attraction up to the spot, and swells the material of which it is formed.*

* This process must be used whenever we have to examine similar spots, whether suspected of containing spermatic fluid, vaginal nasal or urethral mucus, meconium

As soon as the spots were sufficiently distended, we took them off by slightly scraping the cloth with a scalpel; we then placed these scrapings in a drop of the same water previously disposed on a piece of glass, as is generally used for examining objects under the microscope. By means of the ordinary needles used for microscopic preparations, we separated the substance of the crust, which was now of a higher red than in the dry state, and we covered the preparation thus disposed with a thin lamella glass, as usual in microscopic investigation. After having thus settled these preliminary arrangements we placed the preparation under the objective of a microscope magnifying 514 diameters, by means of which we recognize the following facts:

The liquid thns prepared, contained various fragments of the crusts found on the suspected spots, and distended by liquid. These fragments were irregular, some of a greyish appearance, and the others somewhat more colored than the preceding particles.

It was also evident that the liquid around these particles, had taken a reddish tinge, similar to that derived from a dissolution of the coloring matter of the blood. The portion of liquid thus colored formed a red zone of various dimensions around each of the fragments placed under the microscope.

Lastly, both in the liquid thus prepared, and in the very substance of the fragments derived from the spots, delicate microscopic filaments could be seen as if twisted upon themselves, and offering all appearance of cotton filaments; they were all of an indigo color, and contrasted with the pale red color of the liquid.

By adding water while under the microscope to these fragments or crusts of blood, and even before this addition, we could easily ascertain that the fragments now swollen by water were principally composed of fibrin, and secondarily of some of the white globules of blood.

The facts of which we shall next speak could be ascertained:

1. By making use of pure water to swell the spots which were found on the two last strips, out of the four we had taken from the blouse.

2. Or by scraping the small crust as seen under a simple magnifying glass, and receiving it either in the shape of dust or small fragments under the ordinary glass object carrier.

By proceeding in this manner, we found that water discolored the spots or the substance taken up by scraping; that the latter takes a greyish hue, and swells up a little; the water, on the other hand, becomes slightly red, takes up the coloring matter of the red globules of blood, dissolves the colorless elements, and leaves after this action no visible particles behind, such as nucleus or granulations.

When the fragments of the spots thus discolored are separated and broken up by the microscopic needles, and then examined under the microscope, it was found that they were principally formed of a transparent substance, slightly greyish, and finely granular. Moreover, the fragments of this substance, when placed under the microscope, revealed manifestly an arrangement of fibrils with their filaments, straight or slightly waving, intersecting

---

or fœcal matter, See Memoir, by Charles Robin and A. Tardieu, on the microscopical examination of spots formed by meconium or fœtal ceruminous substance, in Annals of Hygiene and Legal Medicine for 1857.

each other, sometimes detached and floating on the sides of the fragments examined.

Having treated these fibrils by acetic acid, they became extremely pale, swelled somewhat, lost their characteristic fibrillar appearance, together with the fine granulations which were scattered through them. Thus from the striated and minutely granular state, they took a homogeneous, transparent and gelatinous appearance.

These attributes are well known to be characteristic of the fibrin of blood, and this appearance is so constant that anatomists can always recognise that important element of blood.

Thus the texture of the smaller crusts or spots submitted to our investigation was entirely composed of fibrin, just as the clot of blood, drawn by venesection, represents one of these crusts on a large scale, beiug formed entirely of fibrin, holding in its meshes the two other solid elements of the blood, the red and white globules of blood.

§ V. *Characters of the white Corpuscles of Blood retained in the fibrin of the Crusts.*

In each fragment of these crusts thus placed under the microscope, the fibrin of which it was composed, after it had been deprived by water of the red globules retained during coagulation, contained several white globules of the blood.

In the substance of this fibrinous texture which we have just described, we found transparent globules of a greyish color, round and finely granular, being from eight to ten thousandths of a millimetre in diameter. In the centre of several of these could be seen one or two small nuclei, greyish, and irregularly spherical or oval, being from three to four thousandths of a millimetre in diameter. Now, such globules in the midst of a fibrinous substance could only be derived from the blood; and, by adding acetic acid to these corpuscles, we discovered all their characteristic reäctions.

We saw the acid gradually rendering each corpuscle more and more transparent, and while swelling these a little, it also destroyed the fine granulations of these globules, and revealed more plainly their nuclei. These soon appeared, two or three in number, and sometimes four, situated towards the centre of the globule, being sometimes near each other in a triangular form, semi-circularly, or one above the other. The acid rendered the edges of these nuclei much darker, more easily seen, precisely as took place in fresh blood examined comparatively: the globules of the blood were also slightly more irregular than they would be from the simple effect of water.

The white globules of blood, whose fundamental characters we thus recognized, were sometimes isolated and disseminated in the fibrinous substance of the clots, sometimes they were contiguous, three or four, and even more being united together side by side.

§ VI. *Special examination of the red globules of the Blood contained in the fibrin of the spots, or adhering to the filaments of the cloth.*

Availing ourselves of the fact that a mixture or solution of different saline substances has the property of preserving the red globules of blood, and restoring them to their original softness after having been dried, we accordingly used these liquids, the advantages of which we had already learned from experience. These liquids are prepared in Paris, by Mr. Burgogne, who is celebrated for his skill in preserving microscopical preparations; but, as a substitute for it, a concentrated solution of sulphate or phosphate of soda, with an addition of ten to fifteen per cent of glycerine, may be used in its stead.

The following is the process by which we succeeded in discovering the characteristic red globules of blood in the spots submitted to our examination. We operated successively on three spots, the diameter of which was from half a millimetre to a millimetre in width. The first set of preparations was made by scraping the slight crust of each spot in a drop of liquid. Covering the whole with a thin slip of glass, we allowed these small dark particles of crust to remain in the liquid during twelve hours. During this maceration, no other precaution is necessary than to protect the preparation from dust, for the liquid being slightly hygrometric, does not evaporate. At the end of this time we perceived that the small particles of crust which were plunged in the liquid had swelled and become more transparent, at the same time that they were of a brighter red than at the commencement of the operation. They had resumed, in fact, all the characteristic color, transparency, consistency and elasticity, which are peculiar to the small masses which are formed under the microscope by the accumulation of the red globules of blood.

By delicately moving the thin plates of glass over each other, an operation familiar to those accustomed to the use of the microscope, we succeeded in detatching from the mass a sufficiently large number of the globules of which it was composed. We were then enabled to observe the characteristics of these globules with as much precision as on fresh human blood.

Each globule had very nearly resumed its original form of a flattened, bi-concave disk. Some, however, still retained the polygonal form, which was due to the compression exercised during their agglomerated condition; others were concave on one side, as in the normal state, but they were convex on the opposite side, as usually happens when the globules of blood are placed in a solution of the sulphate of soda or potash. They measured from six to seven thousandths of a millimetre in width, sometimes a little over; which is the usual size of blood corpuscles. They had resumed their usual reddish yellow tinge, also characteristic of those elements of blood. And lastly, by adding to each preparation one or two drops of acetic acid, we saw these globules gradually becoming paler, until finally they were dissolved in the same manner as those of fresh blood under the same chemical action.

In the preceding examination we were enabled to hasten the swelling and separation of the red globules entering into the composition of the crusts, by adding a little water to the alkaline liquid which we had before employed. For the real object of this liquid is to preserve the elements of fresh blood, and its action on those which have become dry is accordingly slow and sometimes incomplete; but the addition of a small quantity of water, which can be introduced by capillary attraction between the two slips of glass containing the preparation, hastens the separation of the globules, without depriving them of any of their characteristic attributes.

The second series of preparations made from the three previous spots, was executed in the following manner: After having removed the crust from their surface by scraping, there still remained a small spot paler than the other, without any elevation above the tissue of the coat, but having the same form and size as the crust itself. Now, by cutting the surface of the spot thus stained, either with a pair of scissors or a sharp bistoury, and afterwards separating the filaments in a liquid similar to that used for the first preparations, we then allowed the separated filaments to macerate in this liquid. At the end of the same period of time we examined the cotton threads as they appeared in the liquid, and could recognize those characteristics of form and volume presented by the filaments of cotton above described; we also recognized the blue tint of indigo with which the cloth had been dyed, and we, moreover, ascertained that many of these filaments, for a part, or their whole length, were covered with a single coating of red blood globules, or by small red masses formed by the globules of the same kind accumulated and adherent to each other.

We succeeded even more easily with this preparation in separating the red globules of blood, and removing them from the surface of the cotton filaments, by the same process of sliding the glass plates over each other; and their characteristic flattened, bi concave shape, the peculiar color, and reactions with acetic acid were then clearly shown.

Moreover, on most of the filaments we could recognize the globules before they were detached; they appeared somewhat polygonal in shape, by mutual pressure, but they retained their usual size and color, as they formed a coating on the surface of the cotton filaments.

Sometimes the blood corpuscles could be seen on one of their surfaces, sometimes they presented themselves edgeways, at other times they adhered to the filaments by a portion of their surface, while the other projecting outwards, showed a round and flattened disc. Here, again, there could be no doubt: these were evidently red globules, an element which is only found in the blood of a mammiferous animal, being quite different from those of a duck or any other bird. This conclusion was again strengthened by adding an excess of water, or a small quantity of acetic acid to the preparation placed under the microscope, for this agent soon destroyed the red globules which were adherent to the cotton filaments. Those which had been detached, together with the irregular masses formed by these globules, found here and there between the filaments, were seen, as in the normal state, first to swell, and become somewhat paler, and then gradually to disappear as they were dissolved by the liquid.

§ VII. *Examination on the same spot of Blood: 1. Of the fibrin and white Globules. 2. Of the red Globules.*

We proceeded in the following manner with the other spots of blood, one of which was circular, of the size of one millimetre, the other oval, having the same width as the first one, and two millimetres in length.

The crust, of a brownish red, was used for a first series of preparations. For this purpose it was placed for some time in pure water, after having been obtained by scraping: it was then allowed to remain until almost all its color disappeared, and it was then separated with the needles in the manner before described (See Sec. III.) We then examined it with a microscope magnifying 514 diameters, and recognized in those discolored fragments all the characters of fibrin on the one side, and on the other, those of the white globules of blood, such as we have described them above.

We proceeded with these fragments of a brownish red, as with those of the other spots, and found, as we expected, the same corpuscles retained between the meshes of the fibrinous net work. Some of these pieces were placed in the same preservative liquid we used before, and there they retained their red color, and became slightly swollen; but on adding an excess of water in one of the preparations, and a small quantity of acetic acid in the other, these liquids being introduced between the two plates of glass by capillary attraction in the manner above described, the fragments were seen to swell at first, and gradually become paler, until finally they were dissolved under our very eyes. Those which had been treated by water alone, left behind them a thin fibrinous net work surrounded with a circle containing a liquid of a pale yellowish red derived from the coloring matter of the blood dissolved in water.

The small red spot which remained beneath the crust after the latter had been taken from the surface of the cloth, and presenting, therefore, the same form, enabled us to prepare two other specimens; but as the object of these preparations was to examine particularly the red globules of the blood, and not to destroy them with a view of observing the fibrin and white globules, we proceeeded otherwise than before. We therefore used the preservative liquid whose composition we have already given. With a sharp bistoury we scraped away the stained cloth, and then separated those particles in the alkaline liquid, and we examined the filaments under the microscope, after having suffered them to remain for some hours in this same liquid, either pure or diluted with about a tenth part of water. We then recognized the red globules adhering to the surface of the cotton filaments in the manner we have described above (See Sec. VI.)

By again gently moving the two surfaces of glass, one over the other, we could separate some of the red globules from the cotton filaments, and we could thereby ascertain these peculiarities of form, such as the round and biconcave disposition, together with the volume, the color and chemical reactions which we have before described.

It will thus be seen that, by means of the microscope, a single stain can be made to show the existence or absence of the three elements which are especially characteristic of blood. The superficial crust will show the characteristic forms of the fibrin and white globules of blood, while the threads of the cloth immediately beneath and into which the serum of blood carries the red globules, must be used to demonstrate the presence of the red corpuscles.

§ VIII. *Appearance of the corpuscles in the stains formed by the Blood of a Duck.*

In order to answer the question propounded to us in the above commission we caused blood to fall in drops on a blue cotton cloth from the carotid arteries of a duck, by cutting its neck. We left these spots during fifteen days in a dry place, at the ordinary temperature during the month of January, 1857, and we then proceeded to examine them by following exactly the same method observed for the examination of the spots of blood found on the blouse of the accused. By putting the small crusts detached from the spots which were formed by the blood of the duck, in contact with the same alkaline conservative liquid before described, we obtained, at the end of a few hours, a certain number of flattened, oval globules, at least double the size of those of man, and having in the centre a small oblong or ovoid nucleus, quite as charaeteristic of the blood corpuscles of birds, as the lengthened oval shape of the corpuscles themselves.

It is important to observe that the central mark which is seen on the red globules of human blood, and which indicates a central depression existing on both sides of the disk, rendering them in a word bi-concave, is less marked on the globules which have been dried and then softened, than in the fresh state. So that these anatomical elements, after having been softened and separated in the blood stains, do not assume their former bi-concave form, at least not in so well marked a manner as at first. But this circumstance does not prevent them from being recognized by persons familiar with the appearance of the human blood in different conditions.

This small nucleus soon became apparent with clear and distinct outlines under the action of water, and an excess of acetic acid, for these agents constantly produce this effect, by dissolving the body of the globule which has a reddish appearance, but leaving entire the greyish nucleus.

The small crust detached from the stains of the duck's blood were afterwards put into water, and we observed them becoming gradually discolored with a greyish appearance, they likewise remained for some time surrounded by a circle of a pale red color, derived from the coloring matter of the blood corpuscles, dissolved from the small particles thus treated. When this discolcoloration had taken place we did not find the same fibrinous net work, which was manifest in the spots of blood supposed to proceed from the body of a woman. Nothing remained but a considerable number of oblong nuclei of a greyish color, and that which is peculiar to the blood globules of the duck. These nuclei were five or six thousandths of a millimetre in length, by about half of this size in width and thickness. These nuclei were exceedingly close to each other, being for the most part stuck together by a small quantity of colorless matter, in which the characteristiic shreds of fibrin could scarcely be seen. Acetic acid immediately darkened these nuclei, their edges became better defined and somewhat black; at the same time they became more contracted and irregular, as is habitually the effect of this chemical agent on the fresh blood globules.

It was impossible to see the white globules among the mass of nuclei remaining after the action of water and acetic acid on the fragments of the crusts formed by the blood of the duck.

Thus, the oval form and the volume of these globules which was nearly double as compared to those found on the stains of the garment belonging to the accused; the absence of nuclei in the latter, which is always the ease in man, while the presence of ovoid nuclei being constant in the blood of all birds; these characters preclude any possibility of the spots on the blouse being formed by the blood of a duck or any other fowl.

These characteristics enable us easily to recognize human blood in one case, from the blood of the bird, on the other hand, without any possible mistake, for the circular flattened form with no nuclei is as constant in the blood globules of man after the intra uterine period, as the oval flattened form with an oblong nucleus in the blood globules of birds. Moreover, the difference caused by the almost complete absence of the fibrinous shreds in the blood of birds, whilst that of a man contains a large proportion of this substance; the small number or complete absence of white globules in the blood of the first, when compared to their abundant existence in the stains derived from human blood: they are distinctive marks which, though secondary to the first, have yet sufficient value to be attentively examined and noticed in the medico-legal researches.

It may not be unimportant to state that these investigations are among the most delicate which medical jurisprudence may require at rhe hands of the Physician; for these distinctions, though satisfactory and convincing to one accustomed to microscopical research, might not appear equally so to the uninitiated.

§ IX. *Examination of the other Spots on the Blouse, supposed to be of the same origin as the first, though less highly colored, and presumed to have been washed or effaced after their original production.*

We next proceeded to examine by means of the microscope, some of the dust, and small filaments from the cloth of the blouse, which dust was taken from the large stains on the sleeves, on the front and back parts of the blouse with a rusty or brownish appearance, similar to stains of blood after being wiped, or rubbed with earthy matter. We easily ascertained that the small particles of dust which we had detached, as well as those which were still adherent, were composed of small irregular grains of polyhedral shape, sharp angles, with many sides, but presenting no regular arrangement with each other. Some of these grains had no peculiar color, their centre being greyish or almost colorless, with a shining appearance, and thick, dark edges. Their diameter varied faom 5 to 70 thousandths of a millimetre, or even more. Water had no action over them; acetic acid, when added to the former, hardly affected them, except by producing a slight effervescence. Hydrochloric acid alone, dissolved them quite rapidly, with considerable escape of gas. Others of these grains less numerous than the first, presented the same irregular shapes, but a more brilliant red color, resembling somewhat that which is characteristic of the several oxydes and carbonates of iron. The diameter of these dark brown fragments varied from 4 to 35 thousandths of a millimetre: water had no action on them; acetic acid, when added to the preparation, had very little effect, so that in this respect neither of these fragments had properties at all resembling those derived from the spots of blood. The microscopical appearances, which we have just described, being those which are characteristic of the particles of earthy dust, and presenting none of those which are peculiar to the blood, we next examined their real nature and chemical composition by means of the appropriate tests known to science. To attain this object we proceeded as follows: We carefully detached, by means of a sharp bistoury, thirteen spots of a brownish color; some were irregular and large, others were small and round, having from 1 to 3 millimetres in width, and found on the surface of the large reddish spots paler than these particles, as seen on the lower part of the right sleeve. The left sleeve was not touched at all. The same process was repeated on four spots having from one to four millimetres in width, with a reddish brown appearance, taken from the back part of the blouse, and appearing like the first mentioned particles, to be super-imposed or situated over the lighter colored spots beneath them; the front of the blouse was left untouched. The substance thus taken off, when submitted to microscopical investigation, and to the tests already described, presented in due succession the characters of fibrin, with the white globules contained within its meshes, and also the red globules: so that these were exactly similar to the spots which we had already recognized as being formed by blood. The paler spots which remained after taking away the deeper red crusts just described, and presenting the same form and dimensions, did not, however, contain blood globules adhering to the cotton filaments, as we had seen on similar spots found in other parts of this garment.

In these we only found fragments or irregular grains of dust, of a greyish color in some places, in others of a dark red color, such as the rusty spots we have described, which were not formed by the particles of a dark brown color.

After this we carefully scraped, by means of a scalpel, the large rusty colored stains, which were situated on the right sleeve and on the back of the blouse, which stains were of a *lighter color* than the small ones above described.

The dust obtained by scraping these stains, was received in a large porcelain capsule, and then collected together by washing this vessel in warm distilled water. The turbid, muddy liquid thus obtained, was allowed to cool until next morning. By rest it separated into three parts:

1. The portion floating on the surface of the liquid consisted of bluish flakes. Under the microscope it was shown to be composed of cotton filaments, dyed blue, and accompanied with irregular particles, having the appearance of dust particles described at the beginning of this paragraph, and not acted upon by water. These particles were, moreover, in such small quantities, that it was useless, as we shall soon see, to enter into any special examination of their nature.

2. At the bottom of the tube we found a deposit of extremely fine granular powder eight millimetres in depth; the tube itself being about 15 millimetres in diameter. We next decanted the water which had deposited this powder, in order to examine the latter. By microscopical investigation we found that it was entirely composed of irregular corpuscles, some either colorless or of a greyish appearance, others having the dark red appearance of the oxyde or carbonate of iron, such as we have above described; some, which had remained attached to a small cotton fibre, had drawn the latter with it to the bottom. We now lay aside this pulverulent deposit for a subsequent examination, the details of which will be given further on.

3. We next examined the liquid separated by decantation, and which was intermediate between the flakes floating on the surface and the deposit of powder we have just described. The liquid was nearly colorless, having only a greyish tint and slightly turbid. We ascertained, by means of the microscope, that this cloudy appearance was caused by a quantity of short cotton filaments, of a bluish color, which remained in suspension in this liquid. Heated to the boiling point, this liquid was not at all altered; it neither coagulated nor became transparent. We then filtered this liquid, whereupon it became perfectly clear and transparent. Heated over again it remained unaltered. We now put aside this liquid to examine it more particularly, as will be described further on.

From this preliminary examination we had already derived several conclusions, the results of which we will give at once, as they guided us in the choice of the analytical methods which we shall next describe, but which only confirmed these, viz:

1. As the dust obtained from the large brownish spots did not alter in appearance after having remained in water, nor color the water itself to a red or a purplish red, we thus find a corroboration of the microscopical examination, which did not reveal any of the elements of blood in these parcles of powder, for a much smaller quantity of powder obtained from scrap ing the spots of blood would have colored very perceptibly the same quantity of distilled water.

2. The absence of coagulation in the liquid submitted to a temperature of 100°, before and after filteration, shows that it did not contain, in solution, any particles of albuminous matter.

This purely chemical examination had thus shown us, just as the microscope did, that these particles of dust were of mineral origin, entirely foreign to the human body; and therefore the stains were not caused by stains of blood, partially washed or erased by friction.

§ X. *Statement of the Chemical Analysis of the Dust derived from the brownish spots, which, from their color were supposed to be formed by Blood, that had been either washed or rubbed with some substance to make it disappear.*

We first examined the action of distilled water on the dust derived from the stains. In order to do this we divided it into three parts, and put each of these parts into a separate tube. In the first, nitrate of silver gave rise to a decided white, fleecy precipitate, which was dissolved in aqua ammoniæ, thus showing the presence of a small quantity of soluble chlorides.

The chloride of barium produced in the second tube an abundant white precipitate, which was not dissolved when the liquid was slightly acidulated with sulphuric acid. The test revealed the existence of a considerable proportion of soluble sulphates.

In the third portion of the liquid we produced a slight blue color by adding prussiate of potash to its contents, showing traces of the salts of peroxyde of iron. The blue color became somewhat more intense by evaporation and reduction to one-half of the liquid tested.

We then put the pulverulent deposit, of which we have already spoken, into some water, acidulated by hydro-chloric acid; the whole mass was dissolved in the course of about an hour, producing at the same time a continued effervescence and escape of small bubbles of gas. The small quantity of material upon which we operated prevented us from collecting these, but every circumstance goes to prove that they were produced by the action of the hydro-chloric acid upon the carbonates, displacing carbonic acid gas.

The dissolution, when complete, presented a slight bluish tint; the liquid thus obtained was still very acid, as was shown by test paper, which was immediately reddened. We divided it into three equal parts, in as many different tubes. In the first, a small quantity of chloride of barium gave no precipitate; but when the liquor was rendered neutral by the addition of aqua ammoniæ, it immediately gave rise to an abundant white precipitate of phosphate of baryta. A white precipitate was also produced by adding an excess of acetate of lead to another portion of this liquid. These tests revealed to us the prescence of a certain proportion of phosphoric acid, in combination with lime for the most part, forming those irregular grains of mineral dust seen under the microscope, and deposited in distilled water. This will be still better established by the following tests: To the second portion of the liquid we added an excess of oxalate of potash, which immediately produced an abundant granular precipitate, collecting rapidly at the bottom of the tube, and formed of oxalate of lime.

Finally, to the last portion of liquid we added some yellow prussiate of potash, which gave rise to the well-known prussian blue. These different tests thus proved, like the microscopical examination:

1. That the powder derived from these brownish spots—which were somewhat of a lighter color than those in which were seen blood-globules and fibrin—contained none of the elements of blood, nor any other animal matter.

2. That this dust was composed of a small quantity of cotton filaments, but principally of mineral substances, such as those which are found in most of the ordinary earth dusts.

3. That most of the latter substances were in the shape of irregular grains, some greyish, or colorless, formed principally of phosphate and carbonate of lime, with traces of soluble sulphates and chlorides, and perhaps some sulphate of lime.

4. That the other irregular grains, less abundant than the first, and which gave the rusty appearance to the dust, were doubtless composed of the oxyde and carbonate of iron; and the different tests revealed their presence in much greater quantities than those appertaining to animal substances.

5. That the lighter spots, which appear to have been rubbed or spread out by washing, were not produced by blood, but by dust or mud, which had soiled the garment of the accused before the perpetration of the crime. This latter conclusion is corroborated by the fact that on some of these mud stains we found some spots of blood, having the same characteristic appearance as those which were discovered in other parts of this blouse.

### § XI. *Answers to the Interrogatories propounded by the Presiding Judge.*

Having ascertained that the large rusty spots, of a paler color than the others, are not formed by blood, but by earthy matters mixed with oxyde and carbonate of iron in the shape of dust, we can answer:

Yes! the blood from the open carotid artery of a duck may be thrown in sufficient quantity to produce the numerous small bloody stains on the sleeves, the front, and even the back. But, *a fortiori*, these very stains, of whatever shape, could have been derived from arteries situated in different soft parts on the head of an old woman; which arteries may be wounded and cut by violent blows on the head with a sharp or heavy weapon; this conclusion is more probable since an examination of said spots showed evidently that they contained the elements of human blood, and not those of a duck; the experts appointed in this case may now answer the above interrogatories as follows:

1. The dark-red brown stains found on the blouse are formed by blood, which has lost, by evaporation, the water rendering it fluid. In blood alone do we find combined the red globules, the fibrine and white corpuscules, such as we have distinctly recognized in this case.

The microscope alone could have enabled us to decide this question, because those spots were too small to show by chemical tests the albuminous elements of blood. It is, moreover, well known that albumen and its analagous compounds may be found not only in the blood stains, but in many animal liquids, and even in certain vegetable products, whether colored or not. On the contrary, blood alone combines together fibrine, the flattened, circular, red globules, having no nuclei, and the spherical white globules having from one to three nuclei, as revealed by water or acetic acid.

2. Independently of those spots, which, to the naked eye, resemble stains of blood, there were no others of like nature; those which are paler and appear to have been washed or rubbed, are not produced by blood. Their examination showed that they contained none of the elements of blood. For the microscopical examination, completed by the analysis of the substance derived from scraping these spots, showed that they contained grains of mineral and earthy matters, some of a calcareous nature, as the phosphates, carbonates and sulphates; others of a reddish brown color, being composed of the oxydes and carbonate of iron; these substances being generally met with in the mud and dust which produce stains on the clothes of workmen. But on some of these earthy stains certain spots of blood were seen, which had all the characteristic appearances presented on other parts of the blouse not similarly stained.

3. The spots, which are really blood—that is to say, having all its elements, less the water, which has evaporated—do not exist in sufficient quantity for us to say that they could not have been produced by a jet from the arteries of a duck, for these could throw blood far enough to bespatter a person situated near one killing a duck.

4. But the elements of blood forming those stains are not such as are characteristic of the blood of a duck. The blood corpuscles of birds have a flattened, oval shape, a large volume, and a central nucleus.

5. On the other hand, the elements of blood found on these stains unquestionably belong to the human species, for there we find the fibrinous network, and the usual reactions with acetic acid; we find, also, the white corpuscles of human blood having the size, volume, granular appearance, nuclei and chemical reactions which are peculiar to those anatomical elements. The red corpuscles are also distinctly seen, having the size, circular, flattened, bi-concave form, and the reddish hue peculiar to the blood corpuscles of man when seen by transmitted light; they have also the same chemical properties, viz: solubility in water and acetic acid, without leaving any nucleus behind.

But in the present state of the science we can go no farther; we cannot determine from the appearance of the blood, the sex or age of the individual to which it belonged.

6. We can have no hesitation in asserting that these stains could have been derived from a jet of venous or arterial blood caused by wounds on different parts of the head of a person; said wounds being caused by several successive blows from any sharp or heavy instrument.

7. The undersigned experts have shown, by the details with which they have described the different steps of their investigation, that the process to which they had recourse offers greater certainty, exactness and prcision, than any other method heretofere employed. For, the microscope alone, can reveal to us, not the albuminous or ferruginous elements of blood, but its peculiar constituent particles, those by which it is really characterized, in contra-distinction to other liquids of the animal economy, for the fibrin, the red and white globules, are found in blood alone. The certainty and precision of microscopical investigation is proved by the fact, that this instrument alone could have shown whether these spots were formed by human blood, or those of another animal; as it is by means of the microscope that we have established the differences in size, volume and appearance, between human blood globules and those of birds, reptiles and fishes.

Beside the power given to us by this instrument, of examining the smallest possible particles supposed to contain blood, it has also the superior advantage of precision and certainty, for chemical analysis, though operating on large quantities of blood, could only reveal the presence of the albuminous or ferruginous principles of blood; now these elements are common to all the animals above mentioned, as well as to man himself, so that (except by the use of high magnifying powers,) it is utterly impossible to dtscriminate between the blood of a bird and that of a man. Chemical tests are, therefore, of secondary importance, compared to the microscope, in all examinations similar to those above described.—*New Orleans Medical News and Hos. Gazette.*

---

*School of Medicine at Algiers.*—The London Lancet contains an account of a decree dated Aug. 4th, of the present year, for a School of Medicine and Pharmacy, at Algiers. As far as examinations are concerned, the school will be in connection with the Faculty at Montpellier. The diplomas granted in Africa will entitle to practice in the French possessions in any part of the world. It was in Africa, at the Alexandrian school, that public prejudice first gave way and permitted the examination of dead bodies, the greatest boon to the medical profession, inasmuch as it removed the most serious obstacle to its advancement—ignorance of anatomy. Herophilus and Erasistratus were the heads of the Egyptian school, and were the first who were permitted to practice human dissections. These pioneers not only corrected numerous errors, but made many important discoveries. Many of the names given to parts by Herophilus and Erasistratus, still remain in use. Having acquainted themselves with structure, they became bold in their surgical operations. The latter made incisions down to the liver and spleen, and applied medicines directly to these organs.—*Ohio Med. and Surg. Journal.*

